# Psychological Factors Influencing Pain Perception and Experience in Women Undergoing Mammography: A Systematic Review

**DOI:** 10.7759/cureus.98341

**Published:** 2025-12-02

**Authors:** Irene Neophytou, George Charalampous, Eleni Jelastopulu

**Affiliations:** 1 Department of Nursing, Frederick University, Nicosia, CYP; 2 Department of Surgery, Frederick University, Nicosia, CYP; 3 Department of Public Health, University of Patras, Patras, GRC

**Keywords:** anxiety, breast cancer screening, depression, fear of pain, pain, pain catastrophizing, psychological factors, screening mammography

## Abstract

Pain during mammography is a common experience among women and has been identified as a potential barrier to adherence to breast cancer screening programs. While technical and biological factors contribute to pain, emerging evidence suggests that psychological factors may significantly influence pain perception and experience during mammography. This systematic review, therefore, aims to examine the association between psychological factors and the experience of pain during mammography, as well as the intensity of pain experienced during mammography, as measured through validated pain scales or self-reported questionnaires. Preferred Reporting Items for Systematic Reviews and Meta-Analyses Protocol (PRISMA-P) guidelines were used to conduct the systematic review. The electronic databases PubMed and Scopus were used to systematically search for published articles. Keywords used included (mammograph* OR “screening mammograph*” OR mammogram* OR “breast cancer screening” OR “mammographic screening”) AND (“psychological factor*” OR anxiety OR stress OR fear OR “fear of pain” OR pain OR catastrophizing OR “pain catastrophization” OR depression OR “coping strateg*” OR neuroticism OR extraversion) AND (pain OR soreness OR tenderness). Eligibility criteria, including study population, outcome/exposure of interest, study type, language, and publication status, were used to identify relevant literature that measured the association between psychological factors and pain experience. This was a systematic review of publications without year limitations. We found 2,761 articles; 743 were excluded due to duplication. Of the remaining 2,018 studies, 11 were included. Anxiety, pain expectations, previous mammography experience, and nervousness were the most consistently associated psychological factors with increased pain perception during mammography. Coping-related psychological factors showed a consistent pattern: catastrophizing was linked to higher pain intensity, whereas adaptive strategies such as ignoring pain or increasing behavioral activity were associated with less pain. Additionally, higher coping efficacy provided a protective effect on the pain experience. The relationship between depression and pain was inconsistent, and findings regarding personality traits and fear of pain were inconclusive. Pain during mammography was found to be highly prevalent, ranging from 6% to 93% across studies, with moderate to severe pain reported in a substantial proportion of participants. Psychological factors play a significant role in shaping women’s perception and experience of pain during mammography. Anxiety, pain expectations, prior painful experiences, and maladaptive coping strategies such as catastrophizing were associated with higher pain intensity, whereas coping efficacy and adaptive strategies appeared protective. Recognizing these psychological influences is important for understanding differences in women’s mammography pain experiences.

## Introduction and background

Breast cancer remains a major global public health issue, as it is the most common malignancy and one of the leading causes of cancer-related death among women worldwide [1-3]. In 2022, there were 2.3 million new cases of breast cancer and 670,000 deaths worldwide. The incidence rate is increasing by 0.5% annually, with projections suggesting that cases will surpass 3 million by 2040 [3]. Early diagnosis through systematic mammographic screening has been shown to reduce both morbidity and mortality from breast cancer [4,5]. Despite its proven effectiveness, women’s participation in organized mammographic screening programs remains inadequate in many countries, including regions with well-developed healthcare systems [6]. Several factors have been linked to low participation rates, including race or ethnicity, low educational attainment, lack of access to healthcare, absence of a personal or family history of cancer, and low socioeconomic status, as reported in a systematic review by Ponce-Chazarri et al. [7].

**Figure 1:**
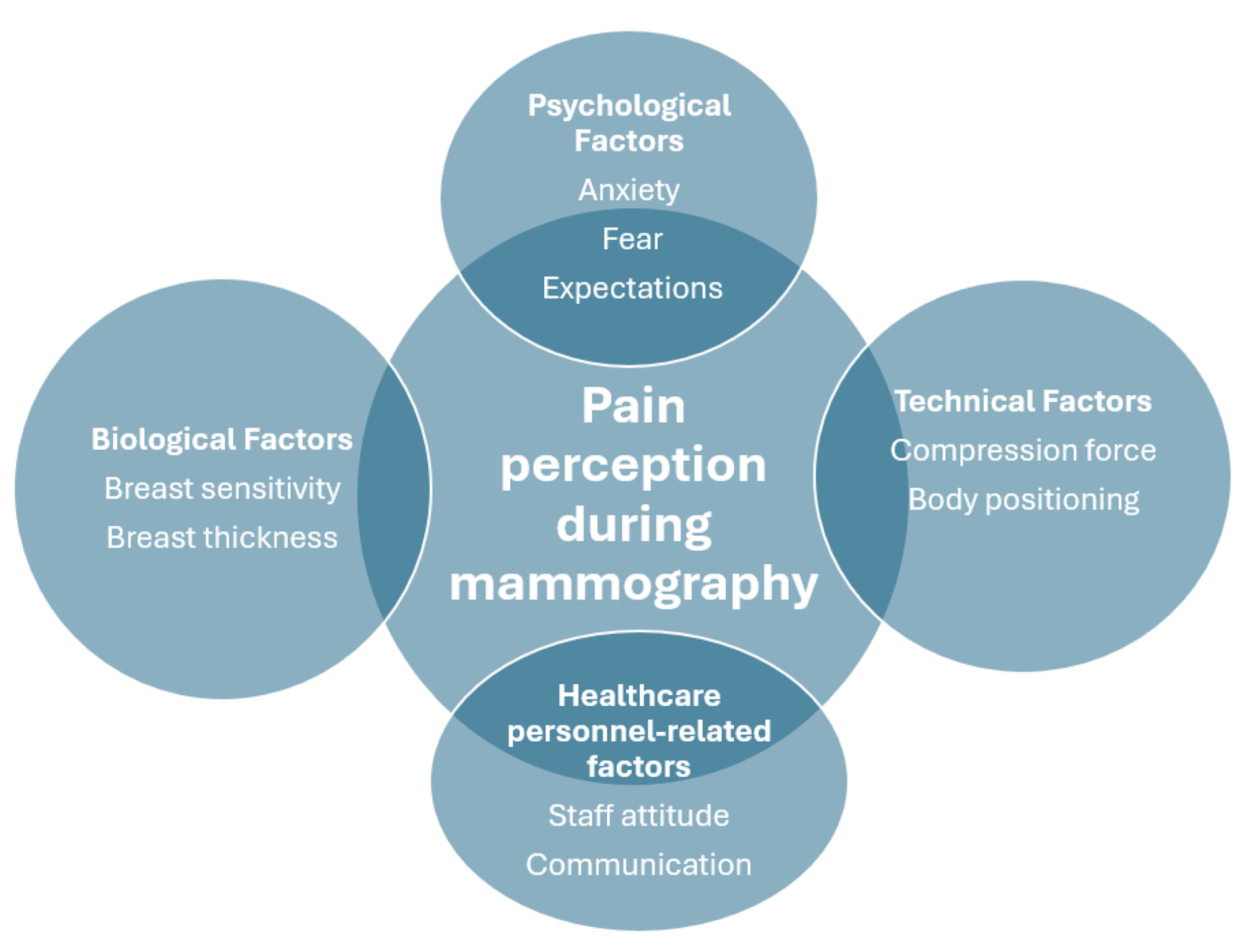
Factors influencing pain perception during mammography

High-quality mammography requires proper breast compression and accurate positioning; however, these procedures can cause pain and discomfort, which may deter women from undergoing future examinations [8-10]. Although pain intensity may depend on biological factors (e.g., breast sensitivity and tissue thickness), technical factors (e.g., compression force and body positioning), and healthcare personnel-related factors (e.g., staff attitude and communication), pain perception is largely subjective and influenced by psychological factors such as anxiety, fear, previous experiences, and expectations (Figure 1) [11,12]. State anxiety and fear of pain are significant psychological factors associated with pain perception [13,14]. Numerous studies have shown that a tendency toward pain catastrophizing, defined as an exaggerated negative cognitive-emotional response to actual or anticipated pain, contributes to greater pain intensity, increased emotional distress, and avoidance of activities that may exacerbate pain [15,16].

In recent years, several interventional studies have aimed to reduce the psychological factors contributing to pain during mammography [17-19]. These approaches have included providing targeted pre-examination information about the procedure, explaining the purpose of breast compression, and offering relaxation or psychoeducational techniques such as breathing exercises, music therapy, and educational videos. While some interventions have reduced anxiety, others have also led to lower reported pain levels [17-19].

Although pain plays a significant role as a deterrent to participating in mammographic screening, no prior systematic review has synthesized the available evidence on multiple psychological factors associated with pain during mammography. To the best of our knowledge, this is the first systematic review to thoroughly examine factors, including anxiety, fear of pain, depression, personality traits, previous experience of mammography, catastrophizing, and coping efficacy, that may influence pain perception and experience during mammography. The aim is to highlight key insights that can inform strategies to reduce psychological burdens and improve adherence to breast screening guidelines.

## Review

Materials and methods

The following research question guided this systematic review: “Is there an association between underlying psychological factors in women undergoing mammography and the experience of pain, and are there any validated pain measurement tools used to assess the intensity of pain experienced during mammography?”

Search Strategy

The review was conducted and reported following the recommendations of Preferred Reporting Items for Systematic Reviews and Meta-Analyses (PRISMA) and was registered with PROSPERO under the number CRD420251117801. A systematic literature search was performed using the PubMed and Scopus databases. The search was limited to these two major databases, and grey literature was excluded. This approach aimed to ensure quality by focusing on peer-reviewed studies; however, it may have reduced the review’s comprehensiveness, as potentially relevant unpublished or non-peer-reviewed research may have been missed.

Search terms were selected using a combination of keywords and Boolean operators (“AND”, “OR”) to create targeted and relevant queries. Keywords included (mammograph* OR “screening mammograph*” OR mammogram* OR “breast cancer screening” OR “mammographic screening”) AND (“psychological factor*” OR anxiety OR stress OR fear OR “fear of pain” OR pain OR catastrophizing OR “pain catastrophization” OR depression OR “coping strateg*” OR neuroticism OR extraversion) AND (pain OR soreness OR tenderness).

This search string represents the full, exact strategy applied to both PubMed and Scopus, with only database-specific syntax differences.

Inclusion and Exclusion Criteria

Studies were selected based on the following eligibility criteria:

Population: Adult women undergoing screening or diagnostic mammography. Studies including women with breast augmentation or those who had undergone breast reconstruction following mastectomy were also eligible. No restrictions were applied based on race or ethnicity; studies reporting on diverse populations were included. A prior history of cancer or the presence of comorbidities was not used as an exclusion criterion.

Exposure of interest: Studies examining psychological factors such as anxiety, depression, fear of pain, coping strategies, expectation of pain, previous experience of pain, nervousness, and personality factors.

Outcomes of interest: The primary outcome was the perception and experience of pain among women undergoing mammography, specifically whether psychological factors influence how pain is perceived and experienced during the procedure. Secondary outcomes included the intensity of pain experienced, as measured by validated pain scales or self-reported questionnaires.

Type of studies: Eligible studies included randomized controlled trials and comparative observational studies, such as prospective and retrospective cohort studies, case-control studies, and cross-sectional studies. Excluded were reviews (narrative or systematic), conference abstracts, opinion articles, letters to the editor, case series, case reports, editorials, theses, and commentaries.

Language and publication status: Only full-text publications in English were included.

Years considered: No restrictions were applied based on publication date.

Study Selection

After removing duplicates, two reviewers (IN and EJ) independently screened titles and abstracts and included eligible articles. Both authors then reviewed full texts for eligibility, resolving any discrepancies through discussion. Titles and abstracts were first screened using the predefined criteria and categorized as eligible or not eligible. In the second step, full texts of potentially eligible studies were thoroughly reviewed for a final decision on inclusion. 

Data Extraction 

We used Rayyan (Rayyan Systems, Inc., Cambridge, Massachusetts, USA), a web-based application designed for systematic reviews, to manage the initial phases of study selection. The platform facilitated the import of records, removal of duplicates, and title and abstract screening. Two reviewers independently screened the studies, with any disagreements resolved through discussion. Subsequently, full-text reading and the final selection of studies for inclusion were conducted using Mendeley Reference Manager, which enabled organized reading, annotation, and reference tracking. A standardized data extraction form was employed to ensure consistency across reviewers and studies. When available, the following data were extracted from each selected study: first author, year of publication, country of study, study design, study setting, sample size, age, participants’ characteristics, psychological factors examined and assessment tools used, pain outcome measures, and main outcomes.

Quality Assessment

The quality of included studies was assessed using JBI critical appraisal tools according to study design [20]. Cross-sectional studies were evaluated with the JBI Critical Appraisal Checklist for Analytical Cross-Sectional Studies (eight items), while cohort studies were assessed with the JBI Critical Appraisal Checklist for Cohort Studies (11 items). Each item was scored as “yes” (one point), “no” or “unclear” (zero points), or “not applicable” (not counted). For each study, a total score and percentage were calculated, and the risk of bias was categorized as high (<50%), moderate (50-70%), or low (>70%). Two authors independently assessed methodological quality, resolving disagreements by consensus; a third reviewer was consulted if consensus could not be reached. No study was excluded based on methodological quality; all were included in the synthesis. This process followed recommendations in the JBI Manual for Evidence Synthesis.

Certainty of Evidence

The Grading of Recommendations, Assessment, Development, and Evaluation (GRADE) approach was applied to determine the overall certainty of the evidence. This framework provides a structured and systematic methodology that considers key factors, including study design, risk of bias, inconsistency, imprecision, and publication bias. Each outcome was assessed according to these domains and classified as providing low, moderate, or high certainty of evidence, ensuring a transparent and reproducible evaluation of the strength of the results. The GRADE approach promoted transparency and consistency in evaluating the overall quality of evidence across outcomes [21].

Data Synthesis and Analysis

The search revealed considerable heterogeneity in study populations, outcome measures, and study designs; therefore, meta-analysis of study findings was not performed. Instead, a structured narrative synthesis was conducted to integrate the findings of the included studies. The synthesis was organized around two main themes. First, associations between psychological factors and pain experience during mammography were examined. Each psychological factor, including depression, anxiety, pain expectation, nervousness, coping strategies, fear, and personality traits, was grouped and synthesized according to its reported association with pain. A thematic narrative was developed to highlight consistent patterns, discrepancies, and gaps across studies. Findings on the association between psychological factors and pain experience were synthesized using both quantitative and qualitative approaches. Extracted quantitative results, such as ORs, correlation coefficients (r), and p-values indicating “significant association” or “no association,” were first classified according to the direction and strength of the association (positive, negative, or nonsignificant). These quantitative findings were then qualitatively coded into broader thematic statements, for example, “anxiety amplifies pain perception.”

Second, the intensity of pain experienced during mammography was examined. Data on pain prevalence and severity were collated from all included studies and summarized to provide an overview of women’s experiences. Reported pain outcomes, including categorical pain levels or prevalence rates, were grouped according to each study’s reporting format. The synthesis emphasized the strength and consistency of associations, methodological limitations, and the relevance of each study’s findings to the review objectives.

Ethical Considerations

Ethical clearance was not applicable, as this review included only published articles from various databases and did not involve human participants.

Results

Study Selection

The primary search of electronic databases PubMed and Scopus initially identified 2,761 studies. After removing duplicates, 743 articles were excluded. Of the remaining 2,018 studies, 1,966 were rejected as irrelevant to the study’s purpose. Ultimately, the systematic review included 11 studies published between 1991 and 2023.

During full-text screening, 41 studies were excluded for various reasons: five were in non-English languages, two were reviews, one was an editorial, one was a letter, 12 had unavailable full texts, three involved participants with breast implants, five reported pain outcomes but did not examine psychological factors, nine were interventional studies, two involved women treated for breast cancer, and one was not limited to mammography screening.

**Figure 2:**
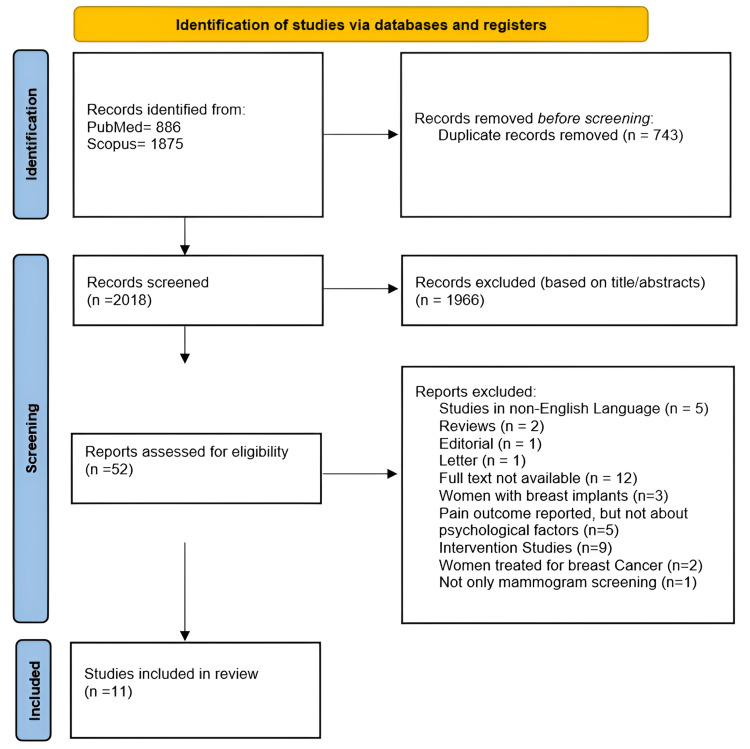
PRISMA study selection flowchart PRISMA, Preferred Reporting Items for Systematic Reviews and Meta-Analyses

The included studies were conducted in Iran (1), the United States (2), the Netherlands (1), Spain (1), the United Kingdom (3), Finland (1), Norway (1), and Belgium (1). Sample sizes ranged from 26 to 1,680 participants, with most studies targeting women aged 40 to 79 years. The complete study selection process is illustrated in Figure 2.

Primary Outcomes

Association between depression and pain experience/perception: Depressive symptoms were examined in two studies, which yielded mixed results. Papas and Klassen (2005) found that depression was positively associated with pain (OR = 1.45; 95% CI: 0.97-2.17; p < 0.10) [22]. In contrast, Aro et al. (1996) did not find a statistically significant association between depressive symptoms and reported pain during mammography [23].

Association between anxiety and pain experience/perception: Anxiety was the most frequently studied psychological factor, assessed in seven of the 11 included studies. Most studies revealed a positive association between anxiety and increased pain during mammography. Keemers-Gels et al. (2000) demonstrated that state anxiety was significantly associated with experienced pain (p = 0.001) [24]. Similarly, Nielsen et al. (1991) reported a strong association between anxiety and mammography-related pain (p < 0.001) [25]. Hafslund (2000) found that anxiety levels were significantly associated with pain after the examination (p < 0.01) [26]. Montoro et al. (2023) evaluated both state anxiety and other psychological characteristics and found that state anxiety was significantly associated with pain intensity during mammography [27]. In contrast, Aro et al. (1996) reported no association between anxiety and pain levels [23].

Association between pain expectation, previous experience, and pain experience/perception: Expectations regarding pain were evaluated in four studies and consistently emerged as strong predictors of experienced pain. Keemers-Gels et al. (2000) and Bruyninckx et al. (1999) found that women who anticipated more intense pain were more likely to report high levels of pain during the procedure (p < 0.05) [24,28]. Rutter et al. (1992) reported that 66% of women who expected mammography to be painful experienced discomfort, compared to only 35% of those who did not anticipate pain [29]. Aro et al. (1996) noted that among women with prior mammography experience, pain expectation was a key predictor of discomfort (r = 0.43, p < 0.001), whereas among women with no prior mammography experience, pain expectation did not have a statistically significant effect [23]. The same study also found that women with earlier mammography experience tended to report more pain (p = 0.269, p < 0.05) [23]. Kornguth et al. (1996) reported a strong positive association between pain experienced during a previous mammogram and pain during the current procedure (McGill Pain Questionnaire = 0.38, Visual Analog Scale (VAS) = 0.41, p < 0.0001) [30].

Association between nervousness and pain experience/perception: Nervousness was evaluated in two studies. Bruyninckx et al. (1999) demonstrated that self-reported nervousness was significantly associated with greater pain perception (p < 0.05) [28]. Similarly, Aro et al. (1996) found that examination-related nervousness was a significant determinant of pain experience, both among women with prior mammography experience (r = 0.41, p < 0.001) and those undergoing mammography for the first time (r = 0.38, p < 0.01) [23].

Association between psychological traits and pain experience/perception: In a study by Montoro et al. (2023) investigating the relationship between psychological traits and pain experience during mammography, extraversion (r = 0.332) and psychoticism showed moderate correlations with pain, but these were not statistically significant (p < 0.05). Neuroticism showed a very weak correlation (r = 0.058, p < 0.05), which was also not statistically significant [27].

Association between fear of pain and pain experience/perception: Montoro et al. (2023) examined the relationship between fear of pain and pain experienced during mammographic screening. No statistically significant positive associations were found between fear of pain and pain intensity across all subscales (r = 0.107-0.248, p < 0.05) [27].

Association between coping strategies and pain experience/perception: Pain-related coping strategies were evaluated in three studies. Asghari and Nicholas (2004) identified significant associations between specific coping strategies and pain intensity. Catastrophizing was positively correlated with pain intensity (r = 0.43), a finding that remained statistically significant after Bonferroni correction (p < 0.003). In contrast, ignoring pain was negatively associated with pain intensity (r = -0.39, p < 0.003). Other strategies, including diverting attention, reinterpreting pain, and praying or hoping, did not show statistically significant associations with pain. Coping self-statements showed a weak negative correlation with pain intensity (r = -0.11, p = 0.11), which did not meet the Bonferroni-adjusted significance threshold (p < 0.003) [31]. Kashikar-Zuck et al. (1997) reported that increasing behavioral activity as a coping strategy was negatively associated with pain intensity (VAS), while other strategies, such as catastrophizing, coping self-statements, diverting attention, ignoring pain sensations, reinterpreting sensations, and praying/hoping, exhibited very weak correlations with pain indicators (p < 0.05) [32]. Montoro et al. (2023) also assessed catastrophizing but did not find a statistically significant association with pain perception (r = 0.109, p < 0.05) [27].

Coping efficacy for pain reduction was significantly associated with lower pain levels across three pain subscales in the study by Kashikar-Zuck et al. (1997), with correlations of r = -0.20 (p = 0.03), r = -0.23 (p = 0.01), and r = -0.27 (p = 0.003) [32]. In contrast, coping efficacy related to pain control was not significantly associated with any pain measures (p > 0.05). In Asghari and Nicholas (2004), belief in one’s ability to reduce pain (pain reduction efficacy) was negatively associated with pain intensity (r = -0.23, p = 0.001), and belief in one’s ability to control pain (pain control efficacy) was associated with lower discomfort levels (r = -0.25, p = 0.001). Both subscales were statistically significant based on the Bonferroni-adjusted threshold of p < 0.003 [31].

Secondary Outcomes

Pain experienced during mammography was a central outcome in most of the included studies. Both the frequency and intensity of pain varied substantially, with reported prevalence ranging from 6% to 93%, reflecting considerable heterogeneity in women’s experiences. In Asghari and Nicholas (2004), 92.3% of participants reported experiencing pain during mammography, with 43.6% describing it as moderate and 10.9% as severe [31]. Similarly, Kashikar-Zuck et al. (1997) found that 74.4% of women experienced pain, with over 30% reporting moderate pain and more than 5% reporting severe pain [32]. Kornguth et al. (1996) reported that up to 91% of participants experienced pain, primarily of mild to moderate intensity, with fewer than 15% reporting severe pain [30].

In Keemers-Gels et al. (2000), 72.9% of women reported pain, categorized as mild (42%), moderate (21.6%), and severe (9.3%), while 27.1% reported no pain [24]. Aro et al. (1996) reported that 61% of participants experienced pain (47% mild, 11% moderate, and 4% severe) [23]. Hafslund (2000) found that 33.5% of women reported no pain, 34.7% reported mild pain, 23.5% moderate, 6.5% strong, and 1.8% intense pain [26]. In contrast, Rutter et al. (1992) recorded a considerably lower frequency, with only 6% of participants reporting pain [29]. Intermediate findings were reported by Nielsen et al. (1991), with 24.2% of women experiencing pain, and by Papas and Klassen (2005), where 76% reported pain and 24% did not [22,25]. Finally, Montoro et al. (2023) reported a statistically significant increase in pain intensity during the mammographic procedure (p < 0.0001), although no specific percentages were provided [27].

The findings of all the aforementioned studies are summarized in Table 1. Instruments and scoring methods for coping strategies across studies are presented in Table 2.

**Table 1:** Characteristics of included studies BDI, Beck Depression Inventory; CES-D, Center for Epidemiologic Studies Depression Scale; CSQ, Coping Strategies Questionnaire; EPQR, Eysenck Personality Questionnaire Revised; FP-III, Fear of Pain Questionnaire, version III; MPQ, McGill Pain Questionnaire; PDRS, Pain Description Rating Scale; STAI, State-Trait Anxiety Inventory; VAS, Visual Analog Scale

Author, year, and country	Study design	Study setting, sample size (N), and age	Participants’ characteristics	Psychological factors examined/assessment tools used	Pain outcome measures	Main outcomes
Papas and Klassen (2005) [22], USA	Cross-sectional study	Low-income urban neighborhoods, N = 530, Y:45-93 (M = 62.3)	Undergoing screening mammogram; 28% had lumpy or cystic breasts; analysis included only women with prior mammography	Depression/CES-D	Self-reported overall pain score (0-4)	Depression associated with pain (OR = 1.45; 95% CI: 0.97-2.17, p < 0.10)
Aro et al. (1996) [23], Finland	Prospective study	Cancer Society Centers & Public or Private Diagnostic Center (Helsinki), N = 1680, Y:50	Undergoing screening mammography. Earlier mammography (N = 684): yes 402, no 282	Anticipation of pain (1 = “not at all”, 2 = “moderately”, 3 = “severely”, 4 = “cannot tell”), Nervousness (1 = “not at all” to 4 = “very”), Earlier mammography, yes/no, Anxiety/STAI, Depression/BDI	Pain measured from 1 = “not at all” to 4 = “severely painful”	Anticipation of pain and discomfort strongly predicted pain in women with prior mammography (r = 0.43, p < 0.001); nervousness also predicted pain (r = 0.41, p < 0.001). In women without prior experience, nervousness was the main predictor (r = 0.38, p < 0.01). Anxiety and depression were not associated with pain. Women with earlier mammography tended to report more pain (p = 0.269, p < 0.05); 61% reported pain: 47% mild, 11% moderate, 4% severe
Keemers-Gels et al. (2000) [24], the Netherlands	Descriptive cross-sectional study	Netherlands breast cancer screening, N = 1200, Y:49.7-75.7 (M = 59.4)	Participating in screening mammography. Menopausal status: premenopausal 15.6%, menopausal 5.6%, postmenopausal 61.9%, hysterectomy 17%	Anxiety (situational variables, yes/no), expectation of pain (“no pain”, “little pain”, “moderate pain”, “severe pain”)	Four-point pain scale (“no pain”, “little pain”, “moderate pain”, “severe pain”)	Expected pain based on prior mammography (p = 0.001) and anxiety (p = 0.001) associated with experienced pain. 72.9% reported pain: mild 42%, moderate 21.6%, severe 9.3%, no pain 27.1%
Nielsen et al. (1991) [25], USA	Cross-sectional study	Sylvester Comprehensive Cancer Center’s Cancer Early Detection Program, N = 272, M = 53.6	Undergoing screening mammography. Menopausal status: premenopausal 30.9%, perimenopausal 2.6%, and postmenopausal 66.4%. Previous mammography: yes 22.4%, no 77.6%	Anxiety/descriptive rating scales	Descriptive Rating Scales, Numeric Rating Scales, VAS	Mammography-related pain showed a statistically significant relationship with anxiety about the mammography experience (p < 0.001, p < 0.05); 24.2% reported pain
Hafslund (2000) [26], Norway	Cross-sectional study	Mammography center in Bergen, N = 170, Y:40-69	Referred for symptomatic mammography	Anxiety/STAI	McGill	Anxiety correlated with pain after mammography (p < 0.01). 33.5% no pain, 34.7% mild, 23.5% moderate, 6.5% severe, 1.8% intense
Montoro et al. (2023) [27], Spain	Cross-sectional study	Centro de Salud Bulevar (Jaen, Andalusia), N = 26, Y:50-69 (M = 58)	Healthy women in routine mammography; no clinical signs of breast pathology or cancer. Menopausal: yes 15.4%, no 84.6%	Anxiety (STAI), Fear of Pain (FP-III), Catastrophizing (CSQ), Psychoticism, Neuroticism, Extraversion (EPQR)	VAS	State anxiety positively associated with pain (r = 0.389, p < 0.05). No significant association for fear of pain (r = 0.213), catastrophizing (r = 0.109), or other psychological traits (r = 0.058-0.332). Significant increase in pain during mammography (p < 0.0001)
Bruyninckx et al. (1999) [28], Belgium	Prospective study	Mobile mammographic screening unit, radiology department of university and non-university hospitals, N = 247, Y:27-78 (M = 54)	Attendance reasons: routine screening 51%, special screening 19%, diagnostic 17%, previous breast cancer treatment 13%, family history 10%. Menopausal status: menopause 8%, post-menopause 62%, menstruating 30%. 74% had prior mammography	Pain expectation and nervousness (nominal or ordinal scale)	10-point scale (0 = no pain, 10 = severe pain)	Pain expectation (p = 0.001) and nervousness (p = 0.010) were significantly associated with pain; ≥73% reported pain
Rutter et al. (1992) [29], UK	Prospective study	Health district in South East Thames region (mobile unit), N = 1160, Y:50-64	Undergoing screening mammography. Menopausal status: premenopausal 22 women; postmenopausal 752 women. Previous mammography: yes 41%, no or unsure 32%	Expectation of pain/interview-based questionnaire post-exam	McGill Pain Inventory	66% of women who expected pain reported discomfort vs 35% who did not; 6% reported pain
Kornguth et al. (1996) [30], UK	Cross-sectional study	Radiology Department of Duke University Medical Center, N = 119, Y:50-79 (M = 61)	Undergoing screening mammography; most subjects postmenopausal; only 5% had never had a mammogram. Excluded women with prior breast cancer or augmentation and any breast symptomatology	Previous mammography experience/self-reported background questionnaire (no validated tool)	McGill, VAS, BPI	Higher pain reported in previous mammography predicted higher pain in the current procedure (MPQ = 0.38, VAS = 0.41, p < 0.0001). Up to 91% reported pain, mostly mild to moderate; <15% severe
Asghari and Nicholas (2004) [31], Iran	Cross-sectional study	Two private radiology departments, Tehran, N = 220, Y:30-71 (M = 47)	Undergoing screening mammography; 61% had no prior mammography; no prior breast cancer, or symptoms	Catastrophizing, Coping Self-Statements, Ignoring Pain Sensations, Diverting Attention, Reinterpreting Pain, Praying/Hoping, Coping Efficacy (CSQ)	VAS, PDRS	Higher catastrophizing associated with higher pain (r = 0.43, p < 0.003). Ignoring pain associated with lower pain (r = -0.39, p < 0.003). Coping efficacy associated with decreased pain (r = -0.23, p < 0.003). 92.3% reported pain: 43.6% moderate, 10.9% severe
Kashikar-Zuck et al. (1997) [32], UK	Cross-sectional study	Radiology Department of Duke University Medical Center, N = 125, Y:50-87 (M = 61)	Undergoing screening mammography; majority (97%) had at least one prior mammogram; mean number of prior mammograms = 9.66; no breast symptoms or prior breast cancer treatment	Increasing Behavioral Activity/CSQ, Catastrophizing/CSQ, Coping Self-Statements/CSQ, Diverting Attention/CSQ, Ignoring Pain Sensations/CSQ, Praying & Hoping/CSQ, Reinterpreting Pain/CSQ, Coping Efficacy/CSQ	100-mm VAS, 6-point PDRS, MPQ, Brief Pain Inventory	High coping efficacy associated with lower pain (r=-0.20, p = 0.03). Individual coping strategies showed weak or no correlations. 93% reported pain; >30% moderate (VAS≥40 or BPI≥4), >5% severe (VAS≥70 or BPI≥7)

**Table 2:** Instruments and scoring methods for coping strategies across studies CSQ, Coping Strategies Questionnaire

Study	Instrument	Subscales	Thresholds
Montoro et al. (2023) Spain [27]	CSQ	Catastrophizing	7-point scale (0-6 per item), total score range 0-36, Cronbach’s α = 0.89. Continuous scoring.
Ashgari and Nicholas (2004) [31], Iran	CSQ	Coping Efficacy (6 scales): Diverting Attention, Reinterpreting Pain Sensations, Coping Self-Statements, Ignoring Pain Sensations, Praying/Hoping, Catastrophizing	Coping Efficacy: Pain decrease control, 7-point scale (0 = can’t decrease at all, 3 = can decrease somewhat, 6 = can decrease completely); Pain control efficacy, 7-point scale (0 = no control, 3 = some control, 6 = complete control). Cronbach’s α: Diverting Attention = 0.79, Reinterpreting Pain Sensations = 0.86, Catastrophizing = 0.70, Coping Self-Statements = 0.76, Ignoring Pain Sensations = 0.70, Praying/Hoping = 0.63. Coping strategies scored on a 7-point scale (0 = never, 3 = sometimes, 6 = always). Continuous scoring.
Kashikar-Zuck et al. (1997) [32], UK	CSQ	Coping Efficacy (7 scales): Diverting Attention, Reinterpreting Pain Sensations, Coping Self-Statements, Ignoring Pain Sensations, Praying/Hoping, Catastrophizing, Increasing Behavioral Activity	Coping Efficacy: Pain decrease control, 7-point scale (0 = can’t decrease at all, 3 = can decrease somewhat, 6 = can decrease completely); Pain control efficacy, 7-point scale (0 = no control, 3 = some control, 6 = complete control). Cronbach’s α: Diverting Attention = 0.85, Reinterpreting Pain Sensations = 0.85, Coping Self-Statements = 0.80, Ignoring Pain Sensations = 0.84, Praying/Hoping = 0.82, Catastrophizing = 0.78, Increasing Behavioral Activity = 0.78. Coping strategies scored on a 7-point scale (0 = never, 3 = sometimes, 6 = always). Continuous scoring.

Risk of Bias Assessment

The methodological quality of the included studies was evaluated using the JBI Critical Appraisal Checklists for cohort and cross-sectional studies. Among the included studies, the percentage of “yes” responses ranged from 62.5% to 87.5%. Overall, most studies were considered to have a moderate risk of bias, while a smaller number were rated as having a low risk of bias. The most common potential sources of bias were inadequate reporting or adjustment for confounding factors, limited methodological detail in describing study settings, and unclear measurement of exposures in some cases. As specified in the review protocol, no study was excluded based on the risk of bias; all studies were included in the analysis regardless of their risk rating. The risk of bias assessments for each study are presented in Table 3 and Table 4.

**Table 3:** JBI Critical Appraisal Checklist for analytical cross-sectional studies Q1: Are the criteria for inclusion in the sample clearly defined?; Q2: Are the study subjects and the setting described in detail?; Q3: Was the exposure measured in a valid and reliable way?; Q4: Were objective, standard criteria used to measure the condition?; Q5: Were confounding factors identified?; Q6: Were strategies to address confounding factors stated?; Q7: Were the outcomes measured in a valid and reliable way?;  Q8: Was an appropriate statistical analysis used? Judgement: Y = Yes, N = No, U = Unclear

Author and year	Q1	Q2	Q3	Q4	Q5	Q6	Q7	Q8	% Yes	Overall risk of bias	Notes
Papas and Klassen (2005) [22]	Y	Y	Y	N	Y	Y	U	Y	62.50%	Moderate	Moderate quality, insufficient confounding control
Keemers-Gels et al. (2000) [24]	Y	Y	N	Y	Y	N	U	Y	62.50%	Moderate	Adequate reporting, limited confounding control
Nielsen et al. (1991) [25]	Y	Y	U	Y	Y	N	U	Y	62.50%	Moderate	Clear methodology, limited confounding control
Hafslund (2000) [26]	U	Y	Y	Y	Y	N	Y	Y	75%	Moderate	Good quality, minor issues in confounding control
Montoro et al. (2023) [27]	Y	Y	Y	Y	Y	N	Y	Y	87.50%	Low	Clear methodology, limited confounding control
Kornguth et al. (1996) [30]	Y	Y	N	Y	Y	Y	U	Y	62.50%	Moderate	Adequate data, insufficient adjustment for confounders
Ashgari and Nicholas (2004) [31]	Y	Y	Y	Y	U	N	Y	Y	75%	Moderate	Clear methodology, limited reporting on confounding
Kashikar-Zuck et al. (1997) [32]	Y	Y	Y	Y	Y	N	Y	Y	87.50%	Low	Clear methodology, limited confounding control

**Table 4:** JBI Critical Appraisal Checklist for analytical cohort studies Q1: Are the two groups similar and recruited from the same population?; Q2: Were the exposures measured similarly to assign participants to both the exposed and unexposed groups?; Q3: Was the exposure measured in a valid and reliable way?; Q4: Were confounding factors identified?; Q5: Were strategies to address confounding factors stated?; Q6: Were the groups/participants free of the outcome at the start of the study (or at the moment of exposure)?; Q7: Were the outcomes measured in a valid and reliable way?; Q8: Was the follow-up time reported and sufficient for outcomes to occur?; Q9: Was follow-up complete, and if not, were the reasons for loss to follow-up described and explored?; Q10: Were strategies to address incomplete follow-up utilized?; Q11: Was an appropriate statistical analysis used? Judgement: Y = Yes, N = No, N/A = Not applicable; U = Unclear

Author and year	Q1	Q2	Q3	Q4	Q5	Q6	Q7	Q8	Q9	Q10	Q11	% Yes	Overall risk of bias	Notes
Aro et al. (1996) [23]	N/A	N/A	Y	Y	Y	Y	N	Y	Y	N	Y	75%	Low	Weaknesses in outcome measurement and handling of incomplete follow-up; clear study design
Bruyninckx et al. (1999) [28]	N/A	N/A	N	Y	Y	Y	N	Y	Y	U	Y	87.50%	Low	Good methodology, limitations in validity and reliability of exposure measurements; unclear handling of incomplete follow-up
Rutter et al. (1992) [29]	N/A	N/A	N	Y	N	Y	Y	Y	Y	Y	Y	62.50%	Moderate	Clear design, no exposure; issues with exposure measurement and confounding factors

Certainty of Evidence

The certainty of the evidence across the included studies was rated as low to very low based on GRADE criteria. For anxiety, findings were consistent, showing that higher anxiety levels correlated with increased pain perception; however, certainty was rated as low due to observational study designs, risk of bias, and potential publication bias. For depression, certainty was very low, primarily because of the small number of studies, inconsistent results, and imprecision. Fear of pain was examined in only one small study, which found no significant association, resulting in a very low certainty rating. Nervousness was studied in two studies that consistently reported links to higher pain, but the limited evidence led to a low certainty rating. Psychological traits were examined sporadically, with no significant associations found, and the certainty was very low. Coping strategies yielded mixed results: catastrophizing was inconsistently linked to higher pain, while ignoring pain and increasing activity were associated with lower pain perception; other strategies showed weak or no consistent findings. Consequently, the certainty of coping strategies was rated very low. Lastly, previous mammography experience appeared to influence pain perception, with women reporting higher pain if they had experienced pain during earlier exams; nevertheless, the certainty of this evidence was considered low due to the limited number of studies and potential bias. Overall, the available evidence suggests possible links between psychological factors, coping strategies, prior mammography experience, and pain perception; however, confidence in these findings is limited, underscoring the need for further high-quality research (Table 5).

**Table 5:** Summary of evidence and certainty of findings according to the GRADE approach GRADE, Grading of Recommendations, Assessment, Development, and Evaluation

Outcome	Studies	Design	No. of participants	Risk of bias	Inconsistency	Indirectness	Imprecision	Publication bias	Effect (narrative)	Certainty	Comment
Anxiety/pain perception	5	3 cross-sectional and 2 prospective	3,348	Serious limitation	No limitation	Minor limitation	No limitation	Serious limitation	Higher anxiety consistently linked with higher reported pain (4/5 studies)	Low	Observational studies, mostly consistent results, but risk of bias and possible publication bias
Depression/pain perception	2	1 cross-sectional and 1 prospective	2,210	Serious limitation	Serious limitation	No limitation	Serious limitation	Very serious limitation	Mixed evidence: one prospective study found an association; one cross-sectional study found no link	Very low	Few studies, inconsistent results, small samples
Fear of pain/pain perception	1	Cross-sectional	26	Minor limitation	Not estimable	No limitation	Very serious limitation	Very serious limitation	No statistically significant association	Very low	Evidence from a single small study
Nervousness/pain perception	2	Prospective	1,927	Serious limitation	Not serious	Not serious	Not serious	Very serious	Both studies found higher nervousness associated with stronger pain experience	Low	Limited number of studies, but consistent evidence
Psychological traits/pain perception	1	Cross-sectional	26	Minor limitation	Not estimable	Not serious	Very serious	Very serious	No statistically significant association	Very low	Evidence from a single small study
Coping strategies/pain perception	3	Cross-sectional	371	Minor limitation	Serious limitation	No limitation	Serious limitation	Very serious	Catastrophizing was inconsistently associated with higher pain; ignoring pain and increasing activity were linked to lower pain; other coping strategies showed weak or no consistent associations	Very low	Observational studies with validated tools, small samples, and inconsistent results
Pain expectation/previous experience	5	2 cross-sectional and 3 prospective	4,412	Serious limitation	No limitation	No limitation	No limitation	Serious limitation	Higher expected pain and previous experience consistently associated with higher pain experience/perception	Low	Large samples and consistent findings, but only 2/5 studies used validated tools

Discussion

To the best of our knowledge, this is the first systematic review to examine the role of psychological factors in the perception and experience of pain among women undergoing mammography. Although systematic reviews exist on individual psychological factors related to pain in other medical contexts, no prior review has specifically focused on women undergoing mammographic examinations.

The first research question aimed to determine whether there is a broadly accepted association between psychological factors and the perception and experience of pain during mammography. The findings of this review indicate that certain psychological factors are statistically associated with increased pain during mammographic screening. These results are interpreted within the broader pain literature, which emphasizes that pain is not merely a physical sensation but a complex experience influenced by biological, social, and psychological factors [33].

Anxiety emerged as the psychological factor most consistently associated with pain experience during mammography, reported in five of the 11 included studies. These findings support the view that anxiety is a significant psychological determinant broadly related to the pain experience. Previous reviews have also concluded that psychological predictors, particularly anxiety, are strongly associated with chronic postoperative pain, further highlighting its relevance in pain-related research [34]. Anxiety and related affective states, such as nervousness, also played a significant role in pain perception during mammography. Nervousness was positively associated with reported pain intensity, regardless of prior mammography experience. This finding aligns with the cognitive-affective model of pain proposed by Eccleston and Crombez (1999), which suggests that negative emotional states, including anxiety and arousal, heighten attention to threatening stimuli and thereby amplify the perception of pain [35]. In contrast, fear of pain was not significantly associated with pain experience in the present review. However, previous systematic reviews have identified fear of pain as an important psychological factor related to pain perception [13].

Pain expectation prior to the examination, as well as previous negative mammography experiences, was identified as a significant predictor of pain experience. This is supported by a previous systematic study that examined the relationship between pain during mammography and re-attendance rates for breast cancer screening. The findings indicated that re-attendance rates were very similar between women who reported pain during mammography and those who did not, suggesting that factors other than pain intensity may influence future screening behavior [36].

Depression was positively associated with pain experience during mammography in only one of the included studies. This may reflect the broader observation that depression is more strongly linked to chronic rather than acute pain. As noted in a related review, patients with chronic pain are at significantly higher risk for depression compared to those without pain [33]. Similar findings have been reported in systematic reviews on chronic pain, such as Bair et al. (2003), who emphasized that depression in patients with pain is associated with greater pain complaints [37]. Moreover, other studies have shown that pain and depression are closely interconnected and may mutually exacerbate physical and psychological symptoms, potentially leading to poorer functional outcomes and prolonged symptom duration [38]. Other researchers have also highlighted that the relationship between depression and pain perception is variable and may depend on the nature of the pain stimulus [39].

The findings of this review highlight the importance of specific pain-coping strategies in the perception of pain during mammography. In particular, catastrophizing emerged as a consistently detrimental factor, showing positive associations with pain intensity [31]. This finding is consistent with previous systematic reviews in postoperative populations, where catastrophizing has been identified as a significant predictor of chronic pain [40]. However, the lack of significant associations between catastrophizing and pain perception in the studies by Montoro et al. (2023) and Kashikar-Zuck et al. (1997) limits the ability to draw generalizable conclusions [27,32]. In contrast, the use of strategies such as ignoring pain and increasing behavioral activity was negatively associated with pain intensity, suggesting a potential protective role.

The concept of coping efficacy, the belief that one can decrease or control pain, shows a consistent association with pain experience, although findings vary across studies. In the study by Kashikar-Zuck et al. (1997), decreased pain efficacy demonstrated statistically significant negative correlations with three pain subscales, suggesting that the more individuals believe they can reduce pain, the lower the reported intensity [32]. In contrast, control pain efficacy was not significantly associated with pain intensity, which may reflect the limited sense of control individuals experience during medical procedures that they cannot modify. Conversely, in the study by Asghari and Nicholas (2004), both dimensions of coping efficacy, the belief that pain can be reduced and the belief that it can be controlled, were significantly associated with lower perceived pain intensity [31].

In the study by Montoro et al. (2023), which analyzed the relationship between personality traits and pain perception during mammography, moderate positive correlations were observed between extraversion and psychoticism and pain intensity [27]. However, these correlations were not statistically significant. Similarly, neuroticism showed a very weak, nonsignificant relationship with pain. These results suggest that while certain personality traits may affect pain experience, their impact on mammography appears limited. This finding contrasts with a previous systematic review, in which psychological traits were shown to influence pain perception during orthodontic treatment [41].

The second research question aimed to examine the level of pain experienced during mammography. The findings of this review indicate that pain during mammographic procedures is highly common, with reported rates reaching as high as 93%. Although in many cases the pain decreases after the exam is over, it cannot be considered temporary or negligible for all women. A large portion of participants report moderate to severe pain. These results align with previous systematic reviews. For example, the review by Whelehan et al. (2013) demonstrated that pain is a widespread and significant obstacle to women’s participation in breast cancer screening, particularly when linked to a previous negative experience [36]. Therefore, pain management should extend beyond technical aspects of the procedure. A comprehensive approach is necessary, including proper preparation, psychological support, clear information, and training of healthcare providers in pain prevention and relief, especially for women with higher anxiety levels or a history of painful mammograms.

A narrative sensitivity analysis was conducted to examine whether the main findings of the present review remained stable when only higher-quality studies, those with a low risk of bias according to the JBI criteria, were considered. The results showed that the associations between anxiety, pain expectations, previous painful experiences, and nervousness with increased pain remained strong even when exclusively analyzing low-risk-of-bias studies. At the same time, coping efficacy and specific adaptive coping strategies, such as ignoring pain and increasing behavioral activity, continued to demonstrate a protective effect. In contrast, the associations involving catastrophizing, depression, fear of pain, and personality traits were less consistent and did not reliably predict the intensity or presence of pain in the high-quality studies.

The wide range in reported pain prevalence (6-93%) can be attributed to a combination of technological, methodological, and cultural factors. Differences in mammography technology (film screen versus digital), screening settings (mobile versus fixed-site units), and procedural techniques likely contribute to this variability. However, most studies did not provide sufficient details on these aspects, making it impossible to perform subgroup analyses based on technological era or screening environment. Other factors that may explain the extensive variation in reported pain prevalence include variability in the pain measurement tools used across studies, cultural differences affecting how pain is experienced or reported, and the timing of pain assessment (e.g., during breast compression versus immediately after the exam). Collectively, these factors likely contribute to the significant heterogeneity observed in pain outcomes.

Limitations and Future Investigation

One of the strengths of this review is its adherence to the PRISMA-P guidelines, which ensure methodological transparency and reproducibility. Moreover, to the best of our knowledge, this is the first systematic review to examine the relationship between psychological factors and pain experience among women undergoing mammography.

However, several limitations should be acknowledged. First, this systematic review does not include a meta-analysis. Studies not published in English were excluded, as were those for which full-text access was not available. Additionally, the search strategy was limited to two databases, and grey literature was excluded, which may have resulted in the omission of relevant studies, including those indexed in other databases and particularly unpublished studies. Publication bias cannot be ruled out, as studies with nonsignificant results may be underrepresented in the published literature. Although no formal assessment of publication bias was conducted, this limitation should be considered when interpreting the findings.

Another important limitation relates to the quality of the included studies: most were observational in design, with small or nonrepresentative samples, and some relied on self-reported measures, which increases the risk of bias. According to the GRADE framework, the certainty of evidence was rated as low to very low across all outcomes, indicating that the observed associations should be interpreted with caution. A major limitation of this review is the considerable heterogeneity among studies, reflected in the wide range of reported pain prevalence (6-93%). Although the sensitivity analysis supported the robustness of the main findings, variations in mammography technology, pain measurement methods, cultural factors, and assessment timing likely contributed to inconsistencies. Furthermore, insufficient methodological detail in several studies limited the ability to conduct subgroup analyses. These issues underscore the need for more rigorous and consistently reported research in the future.

This review revealed limited and nonsignificant associations between personality traits, coping strategies, and pain perception during mammography, as well as heterogeneity in the coping strategies related to pain. Therefore, further investigation into the relationship between personality traits, coping strategies, and pain perception in the context of mammographic screening is warranted. There was also substantial heterogeneity in the instruments used to assess pain and psychological factors. The use of standardized and validated questionnaires is recommended to enhance comparability across studies and strengthen the validity of the conclusions. Furthermore, a qualitative research approach could offer a deeper understanding of women’s subjective pain experiences, particularly among those with psychological vulnerability, thereby supporting the development of more targeted and personalized interventions.

Practical Recommendations

Given that most available evidence is observational, practical recommendations should be made with caution. However, our findings suggest that a brief anxiety screening, followed by targeted radiology technologist-led counseling for women with high anxiety, could be a practical and effective approach, as anxiety is consistently a key predictor of pain. Universal multimedia education could also serve as a low-cost supplementary method to improve preparedness and reduce distress. Further experimental research is needed to identify the most effective combination of interventions.

## Conclusions

This systematic review highlights the crucial role of psychological factors in shaping women’s perceptions and experiences of pain during mammography. Anxiety, pain expectations, previous mammography experiences, nervousness, and specific coping strategies, particularly catastrophizing, were associated with higher pain levels. Conversely, coping efficacy and strategies such as ignoring pain and increasing behavioral activity appeared to have a protective effect. Although results regarding personality traits and fear of pain were inconsistent, this review underscores that pain is a multidimensional experience influenced not only by physiological and technical factors but also by the psychological state and cognitive processes of the individual undergoing the procedure.

Because pain can discourage participation in breast cancer screening, recognizing the influence of psychological factors can help guide future strategies aimed at improving women’s comfort and supporting continued participation in screening programs. While these findings provide valuable insights, they should be interpreted in light of the methodological limitations of the included studies, most of which employed observational designs and presented a moderate risk of bias, potentially affecting the overall strength and certainty of the evidence.

## References

[1] Coleman C (2017). Early detection and screening for breast cancer. Semin Oncol Nurs.

[2] Harbeck N, Gnant M (2017). Breast cancer. Lancet.

[3] Xu H, Xu B (2023). Breast cancer: epidemiology, risk factors and screening. Chin J Cancer Res.

[4] Marmot MG, Altman DG, Cameron DA, Dewar JA, Thompson SG, Wilcox M (2013). The benefits and harms of breast cancer screening: an independent review. Br J Cancer.

[5] Freeman K, Geppert J, Stinton C, Todkill D, Johnson S, Clarke A, Taylor-Phillips S (2021). Use of artificial intelligence for image analysis in breast cancer screening programmes: systematic review of test accuracy. BMJ.

[6] Mandal R, Basu P (2018). Cancer screening and early diagnosis in low and middle income countries : current situation and future perspectives. Bundesgesundheitsblatt Gesundheitsforschung Gesundheitsschutz.

[7] Ponce-Chazarri L, Ponce-Blandón JA, Immordino P, Giordano A, Morales F (2023). Barriers to breast cancer-screening adherence in vulnerable populations. Cancers (Basel).

[8] Keefe FJ, Hauck ER, Egert J, Rimer B, Kornguth P (1994). Mammography pain and discomfort: a cognitive-behavioral perspective. Pain.

[9] Moshina N, Sebuødegård S, Evensen KT, Hantho C, Iden KA, Hofvind S (2019). Breast compression and experienced pain during mammography by use of three different compression paddles. Eur J Radiol.

[10] Gupta R, Nayak M, Khoursheed M, Roy S, Behbehani AI (2003). Pain during mammography: impact of breast pathologies and demographic factors. Med Princ Pract.

[11] Davey B (2007). Pain during mammography: possible risk factors and ways to alleviate pain. Radiography.

[12] Akansel N, Gülşen M, Gültaş M (2021). Influence of discomfort tolerance of women who undergo mammography on the perceived pain intensity due to the procedure. Eur J Breast Health.

[13] Markfelder T, Pauli P (2020). Fear of pain and pain intensity: meta-analysis and systematic review. Psychol Bull.

[14] Tang J, Gibson SJ (2005). A psychophysical evaluation of the relationship between trait anxiety, pain perception, and induced state anxiety. J Pain.

[15] Sullivan MJ, Rodgers WM, Wilson PM, Bell GJ, Murray TC, Fraser SN (2002). An experimental investigation of the relation between catastrophizing and activity intolerance. Pain.

[16] Sullivan MJ, Thorn B, Haythornthwaite JA, Keefe F, Martin M, Bradley LA, Lefebvre JC (2001). Theoretical perspectives on the relation between catastrophizing and pain. Clin J Pain.

[17] Fernández-Feito A, Lana A, Cabello-Gutiérrez L, Franco-Correia S, Baldonedo-Cernuda R, Mosteiro-Díaz P (2015). Face-to-face information and emotional support from trained nurses reduce pain during screening mammography: results from a randomized controlled trial. Pain Manag Nurs.

[18] Zavotsky KE, Banavage A, James P, Easter K, Pontieri-Lewis V, Lutwin L (2014). The effects of music on pain and anxiety during screening mammography. Clin J Oncol Nurs.

[19] Kuo CP, Li PC, Chuang HL, Lee SH, Liao WC, Lee MS (2021). The effect of multimedia health education on pain and anxiety in women undergoing mammography in Taiwan. Taiwan J Obstet Gynecol.

[20] Joanna Briggs Institute (JBI (2025). Critical appraisal tools. https://jbi.global/critical-appraisal-tools.

[21] Prasad M (2024). Introduction to the GRADE tool for rating certainty in evidence and recommendations. Clin Epidemiol Glob Health.

[22] Papas MA, Klassen AC (2005). Pain and discomfort associated with mammography among urban low-income African-American women. J Community Health.

[23] Aro AR, Absetz-Ylöstalo P, Eerola T, Pamilo M, Lönnqvist J (1996). Pain and discomfort during mammography. Eur J Cancer.

[24] Keemers-Gels ME, Groenendijk RP, van den Heuvel JH, Boetes C, Peer PG, Wobbes TH (2000). Pain experienced by women attending breast cancer screening. Breast Cancer Res Treat.

[25] Nielsen BB, Miaskowski C, Dibble SL, Beber B, Altman N, McCoy CB (1991). Pain and discomfort associated with film-screen mammography. J Natl Cancer Inst.

[26] Hafslund B (2000). Mammography and the experience of pain and anxiety. Radiography.

[27] Montoro CI, Alcaraz MD, Galvez-Sánchez CM (2023). Experience of pain and unpleasantness during mammography screening: a cross-sectional study on the roles of emotional, cognitive, and personality factors. Behav Sci (Basel).

[28] Bruyninckx E, Mortelmans D, Van Goethem M, Van Hove E (1999). Risk factors of pain in mammographic screening. Soc Sci Med.

[29] Rutter DR, Calnan M, Vaile MS, Field S, Wade KA (1992). Discomfort and pain during mammography: description, prediction, and prevention. BMJ.

[30] Kornguth PJ, Keefe FJ, Conaway MR (1996). Pain during mammography: characteristics and relationship to demographic and medical variables. Pain.

[31] Asghari A, Nicholas MK (2004). Pain during mammography: the role of coping strategies. Pain.

[32] Kashikar-Zuck S, Keefe FJ, Kornguth P, Beaupre P, Holzberg A, Delong D (1997). Pain coping and the pain experience during mammography: a preliminary study. Pain.

[33] Mee S, Bunney BG, Reist C, Potkin SG, Bunney WE (2006). Psychological pain: a review of evidence. J Psychiatr Res.

[34] Giusti EM, Lacerenza M, Manzoni GM, Castelnuovo G (2021). Psychological and psychosocial predictors of chronic postsurgical pain: a systematic review and meta-analysis. Pain.

[35] Eccleston C, Crombez G (1999). Pain demands attention: a cognitive-affective model of the interruptive function of pain. Psychol Bull.

[36] Whelehan P, Evans A, Wells M, Macgillivray S (2013). The effect of mammography pain on repeat participation in breast cancer screening: a systematic review. Breast.

[37] Bair MJ, Robinson RL, Katon W, Kroenke K (2003). Depression and pain comorbidity: a literature review. Arch Intern Med.

[38] IsHak WW, Wen RY, Naghdechi L (2018). Pain and depression: a systematic review. Harv Rev Psychiatry.

[39] Thompson T, Correll CU, Gallop K, Vancampfort D, Stubbs B (2016). Is pain perception altered in people with depression? A systematic review and meta-analysis of experimental pain research. J Pain.

[40] Burns LC, Ritvo SE, Ferguson MK, Clarke H, Seltzer Z, Katz J (2015). Pain catastrophizing as a risk factor for chronic pain after total knee arthroplasty: a systematic review. J Pain Res.

[41] Lorek M, Jarząbek A, Sycińska-Dziarnowska M (2024). The association between patients' personality traits and pain perception during orthodontic treatment: a systematic review. Front Neurol.

